# Curative efficiency and adverse events of alternative therapy and medicine for functional constipation in adults: A protocol for systematic review and meta-analysis

**DOI:** 10.1097/MD.0000000000029082

**Published:** 2022-04-08

**Authors:** Chang Liu, Tingting Pang, Shuang Yin, Jiahui Li, Junjie Yao, Hongmei Li, Huijuan Lou, Siyuan Lei, Jiangchun Zhang, Li Dong, Yufeng Wang

**Affiliations:** a Department of Acupuncture and Tuina, Changchun University of Chinese Medicine, Changchun, China; b Department of Rehabilitation Medicine, Changchun University of Chinese Medicine, Changchun, China; c Department of Tuina, the Affiliated Hospital to Changchun University of Chinese Medicine, China.

**Keywords:** acupuncture, functional constipation, laxative, massage, meta-analysis

## Abstract

**Background:**

The efficacy of alternative therapies and medications for functional constipation (FC) in adults is well established, however, the efficacy and safety of different alternative therapies and medications for FC in adults is not fully clarified. Due to there are many different alternative therapies and medications available for the treatment of febrile FC in adults, the selection of appropriate alternative therapies and medications has become an urgent issue. The purpose of this study was to evaluate the effectiveness and safety of alternative therapy and medicine for FC in adults.

**Methods:**

PubMed, Web of Science, Scopus, Cochrane Library, Embase, China National Knowledge Infrastructure, China Science and Technology Journal Database and Wanfang Data were searched to identify randomized controlled trials which focused on alternative therapy and medicine for FC in adults from December 15, 2020 to July 1, 2021. Subsequently, 2 researchers will be independently responsible for literature screening, data extraction, and assessment of their quality. This study uses The R Programming Language 4.0.2 based on Bayesian framework for NMA. Odds ratios or standardized mean differences will be modeled using Markov chain Monte Carlo methods, both with 95% confidence intervals.

**Results:**

The results of this meta-analysis will be submitted to a peer-reviewed journal for publication.

**Conclusions:**

The conclusion of this systematic review will provide evidence for selecting an optimal alternative therapy and medicine for patients with FC in adults.

**Ethics and dissemination:**

The protocol of the systematic review does not require ethical approval because it does not involve humans. This article will be published in peer-reviewed journals and presented at relevant conferences.

**Systematic review registration:**

INPLASY202210091.

## 1 Introduction

Functional constipation (FC) is a common gastrointestinal disorder with the main clinical manifestations being difficult bowel movements, hard or lumpy stools, and thinning.^[1]^ The prevalence of FC has been reported to be around 20% of adults in the USA and 14% of adults in the UK.^[2,3]^ FC poses a significant clinical and financial burden to patients.^[4]^ For a small proportion of patients, changes in lifestyle habits, such as a vegetarian diet or increased aerobic activity, can alleviate their symptoms. Most patients, however, also require medical treatment.^[5,6]^ Currently, the treatments used include gastrointestinal stimulants, enemas, osmotic agents and stimulant laxatives. There is some efficacy in FC. However, after prolonged use of these treatments, many side effects can occur.^[7]^ Therefore, a safe and effective treatment for FC has been desired.^[8]^

Complementary and alternative medicine (CAM) is often used to treat chronic diseases as well as for disease prevention.^[9,10]^ In China and some other Asian countries, acupuncture and abdominal tuina have been used as a form of CAM for the treatment of gastrointestinal disorders for approximately 3000 years.^[11–13]^ Studies have shown that acupuncture can modulate gastrointestinal motility and acid secretion^[14,15]^; electro-acupuncture can alter the motility of the gastrointestinal tract^[16]^; and abdominal tuina can induce rectal muscle waves, stimulate somatic autonomic reflexes and intestinal sensation, and promote rectal loading and peristalsis.^[17]^ In recent years, a growing number of clinical studies have used CAM to intervene in FC. Although many clinical studies have reported a positive effect on FC, there is no scientific evidence. Therefore, the aim of this mesh meta-analysis is to evaluate the efficacy and poor prognosis of CAM and pharmacological treatment of FC to provide a better basis for clinical decision making.

## 2 Methods

### 2.1 Study registration

The protocol of this review was registered in INPLASY (Registration: INPLASY202210091). Besides, it was reported as per the statement guidelines of preferred reporting items for systematic reviews and meta-analysis protocol.

### 2.2 Inclusion criteria for study selection

#### 2.2.1 Types of studies

Only randomized controlled trials (RCTs) investigating the efficacy and safety of alternative therapy and medicine for FC in adults will be included in this study.

#### 2.2.2 Types of participants

All patients should be diagnosed with FC and show symptoms of reduced frequency of bowel movements and difficulty in passing stools, and should be older than 18 years of age. However, race, gender, and educational status are not limited. The diagnosis of FC should meet the Rome IV Criteria.^[18]^ Patients with other organic diseases will be excluded.

#### 2.2.3 Types of interventions

The intervention in the experimental group should be choose alternative therapy or medicine, and the interventions of the control group should only be no treatment, sham or placebo groups, or other conventional treatments. The methods of alternative therapy include acupuncture or massage. The medicine include lactulose, polyethylene glycol, milk of magnesia, mineral oil, bisacodyl, senna, sodium picosulfate, bisacodyl, sodium phosphate, sodium docusate, sodium lauryl sulfoacetate, probiotics, lubiprostone, linaclotide, plecanatide, and prucalopride.

#### 2.2.4 Types of outcome indexes

1) The primary outcome was frequency and quantity of defecation. Only a single treatment regimen can be used in the intervention group or control group. The sixth week will be selected as the observation node of efficacy. If there are multiple observation nodes of efficacy, the node nearest to the time of the sixth week will be selected for observation. There was no statistical difference in the corresponding data of all subjects before the test.
2) Secondary endpoints included faecal incontinence, disimpaction, need for additional therapies, and adverse events.
3) Measures of effect include changes in other gastrointestinal symptoms experienced by the patient during treatment and degree of side effects to the treatment.

### 2.3 Exclusion criteria

(1) Abstracts or full text not available.
(2) Studies for which data could not be extracted accurately.
(3) Repeatedly published studies were reported with data selected from the most comprehensive information and the longest follow-up.
(4) Studies with inconsistent outcome indicators.

### 2.4 Data sources

All RCTs investigating the efficacy and safety of alternative therapy and medicine for FC in adults published before April 30, 2021 will be systematically searched from PubMed, Web of Science, Scopus, Cochrane Library, Embase, China National Knowledge Infrastructure, China Science and Technology Journal Database, and Wanfang Data. The reference lists of all retrieved articles will also be manually reviewed, with the aim of identifying any relevant trails. In the light of different electronic databases, the search terms and search strategy in this study will be adjusted correspondingly, which will conduce to avoiding the problem of mismatching. The details of PubMed search strategies are illustrated in Table 1.

**Table 1:** Search strategy used in PubMed.

No	Search items
**#1**	Constipation [Mesh]
**#2**	Functional constipation [Title/Abstract] OR constipation [Title/Abstract]
**#3**	#1 OR #2
**#4**	Lactulose [Title/Abstract] OR polyethylene glycol OR milk of magnesia [Title/Abstract] OR mineral oil [Title/Abstract] OR bisacodyl [Title/Abstract] OR senna [Title/Abstract] OR sodium picosulfate [Title/Abstract] OR bisacodyl [Title/Abstract] OR sodium phosphate [Title/Abstract] OR sodium docusate [Title/Abstract] OR sodium lauryl sulfoacetate [Title/Abstract] OR probiotics [Title/Abstract] OR lubiprostone [Title/Abstract] OR linaclotide [Title/Abstract] OR plecanatide [Title/Abstract] OR prucalopride [Title/Abstract]
	Acupuncture [Title/Abstract] OR tuina [Title/Abstract] OR massage[Title/Abstract]
**#5**	#4 OR #5
	(randomized controlled trial[pt] OR controlled clinical trial[pt] OR randomized[tiab] OR placebo[tiab] OR clinical trials as topic [mesh:noexp] OR randomly[tiab] OR trial[ti])NOT (animals[mh] NOT humans[mh])
**#6**	adult[Mesh]
**#7**	Aged[Title/Abstract]OR young adult[Title/Abstract] OR middle aged[Title/Abstract] OR frail elderly[Title/Abstract]
**#8**	#8 OR #9
**#9**	#3 AND #6 AND #7 AND #10
**#10**	
**#11**	

### 2.5 Data collection and analysis

#### 2.5.1 Data extraction and management

**Figure 1:**
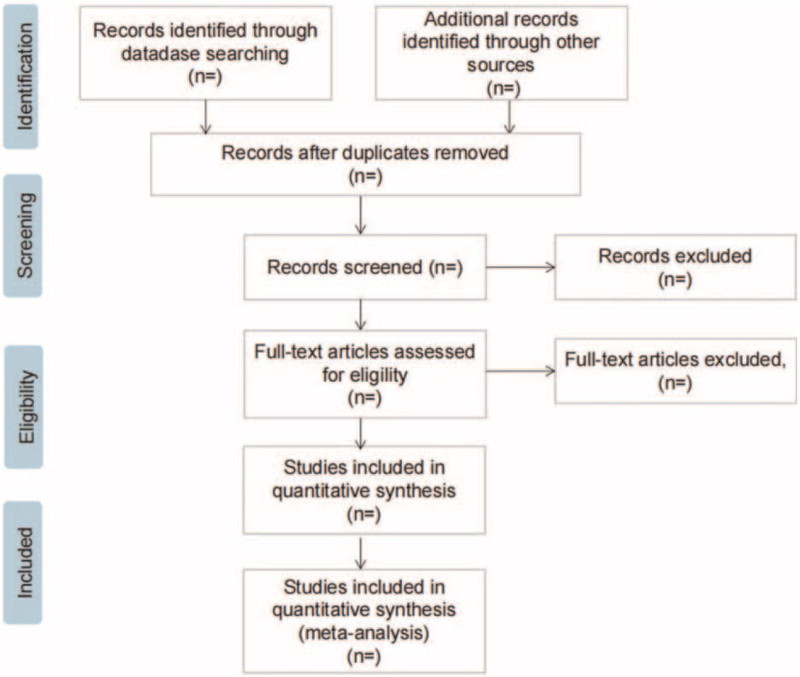
Flow diagram of study selection process.

The data will be extracted out by 2 independent reviewers in accordance with the standardized sheet recommended by the Cochrane Handbook of Systematic Reviews of Interventions. The extraction contents contain: RCT characteristics: title, name of the first author, publication date, literature sources, and quality evaluation items of RCTs. Baseline characteristics of patients: size, age, gender, tumor types, tumor stages, and so forth. Intervention: the specific content, modalities and operational specifications of the intervention, the people involved, the timing, frequency and periodicity of the intervention, and whether the people involved in the intervention receive training. Outcomes: frequency and quantity of defecation, change in the number of weekly bowel movements and the number of lumpy or hard stools in per bowel movement, faecal incontinence, disimpaction, need for additional therapies and adverse events, changes in other gastrointestinal symptoms experienced by the patient during treatment and degree of side effects to the treatment, etc. If there is any inconsistent opinion, it will be further negotiated and arbitrated with a third researcher. The screening flow chart of this study is presented in Figure 1.

#### 2.5.2 Assessment of risk of bias

Two evaluators will independently evaluate the quality of the included RCTs with the Cochrane risk of bias tool (Cochrane Handbook, version 5.1.0).^[19]^ The evaluation results will be classified into the high-risk, low-risk, and unclear categories.

#### 2.5.3 Measures of treatment effect

For dichotomous outcomes, the risk ratio will be used in the meta-analysis. All of these data will be summarized with a 95% confidence interval (CI). The survival data will be expressed with hazard ratios and 95% CI.

#### 2.5.4 Management of missing data

In case of any missing data in relevant study, the original data will be requested by email. If there is a failure in the data request, such data shall be excluded from this study.

#### 2.5.5 Assessment of heterogeneity and data synthesis

The R Programming Language 4.0.2 software will be used to perform the pairwise network meta-analysis. Odds ratios or standardized mean differences will be modeled using Markov chain Monte Carlo methods, both with 95% CIs. Preset model parameters: 4 chains are used for simulation analysis, with an initial value of 2.5, a step size of 20,10,000 annealing times, and 50,000 simulation iterations. The network evidence plot will be generated according to different outcome. According to the results of the NMA, rank probability plot of various CAM therapies will be generated and sorted by dominance, with Rank 1 being the optimal sort.

##### 2.5.5.1 Heterogeneity test

Before the combination of effect size, we will use The R Programming Language 4.0.2 to assess available study and patient characteristics to ensure similarity and to investigate the potential effect of heterogeneity on effect estimates. When interstudy heterogeneity exists, the random effect model is used. For comparison of each pair, heterogeneity is assessed by the statistic value. When greater than 50%, it indicates that there is heterogeneity between studies, and the source of heterogeneity should be further searched. When less than 50%, interstudy heterogeneity is considered to be small or there is no obvious heterogeneity.

#### 2.5.6 Assessment of reporting biases

A funnel plot will be performed to analyze the existence of publication bias if 10 or more pieces of literature are included in this meta-analysis.^[20]^

#### 2.5.7 Subgroup analysis

If necessary, we will conduct a subgroup analysis of the different doses of the drug, treatment time, and different subtypes of FC.

#### 2.5.8 Sensitivity analysis

The sensitivity analysis will be conducted to assess the reliability by excluding each study each time and calculating the remaining.

#### 2.5.9 Grading the quality of evidence

The grading of recommendations assessment, development, and evaluation will be adopted to evaluate the quality of evidence from the following 5 aspects: risk of bias, indirectness, inconsistency, imprecision, and publication bias.^[21]^ The quality of evidence will be graded as high, moderate, low, and very low.

#### 2.5.10 Ethics and dissemination

The contents of this paper do not involve moral approval or ethical review and will be presented in print or at relevant conferences.

## 3 Discussion

FC is a common gastrointestinal disorder with a high incidence and a positive correlation with age.^[22]^ Chronic constipation is extremely harmful to a person's body. Patients are not only prone to gastrointestinal dysfunction, haemorrhoids, anal fissures, insomnia and anxiety, but also increase the chances of colorectal carcinoma and the risk of cardiovascular disease.^[23,24]^ In addition, the disease has a high recurrence rate, which places a heavy financial burden on patients. Therefore, it is particularly important to choose a good treatment method.^[25]^

Currently, pharmacological and alternative treatments are commonly used in clinical practice. The main drugs used are dietary fibre supplements, stool softeners, osmotic and stimulant laxatives and motivational agents.^[26]^ Alternative therapies are mainly acupuncture and tui na. Both approaches are each recognised by patients, but it is unclear who has the greater efficacy and safety advantages. This study will summarise and rank the efficacy and safety of alternative therapies and medications used to treat FC to provide a reference for determining the best treatment method.

## Author contributions

Yufeng Wang contributed to the conception of the study. Yufeng Wang and Chang Liu drafted and revised the manuscript. The search strategy was developed by all the authors and will be performed by Chang Liu, Tingting Pang, Jiahui Li, and Junjie Yao will independently screen the potential studies and extract data from the included studies. The formal analysis will be done by Yin Shuang and Huijuan Lou. Assess the risk of bias and complete Siyuan Lei, Jiangchun Zhang, and Hongmei Li. Chang Liu will complete data synthesis. Li Dong arbitrate disagreement, Yufeng Wang ensured the absence of any errors. All authors approved the publication of the protocol.

**Conceptualization:** Chang Liu, Yufeng Wang.

**Data curation:** Chang Liu, Tingting Pang, Jiahui Li, Junjie Yao.

**Formal analysis:** Huijuan Lou.

**Funding acquisition:** Shuang Yin.

**Investigation:** Shuang Yin.

**Methodology:** Shuang Yin.

**Project administration:** Yufeng Wang.

**Resources:** Tingting Pang, Shuang Yin.

**Software:** Shuang Yin.

**Supervision:** Li Dong.

**Validation:** Siyuan Lei.

**Visualization:** Hongmei Li, Jiangchun Zhang.

**Writing – original draft:** Chang Liu, Yufeng Wang.

**Writing – review & editing:** Chang Liu, Yufeng Wang.
